# Lack of Melanopsin Is Associated with Extreme Weight Loss in Mice upon Dietary Challenge

**DOI:** 10.1371/journal.pone.0127031

**Published:** 2015-05-26

**Authors:** Didem Göz Aytürk, Ana Maria Castrucci, David E. Carr, Susanna R. Keller, Ignacio Provencio

**Affiliations:** 1 Department of Biology, University of Virginia, Charlottesville, Virginia, United States of America; 2 Department of Environmental Sciences, University of Virginia, Charlottesville, Virginia, United States of America; 3 Department of Medicine—Division of Endocrinology, University of Virginia School of Medicine, Charlottesville, Virginia, United States of America; 4 Departamento de Fisiologia, Universidade de São Paulo, São Paulo, Brasil; Morehouse School of Medicine, UNITED STATES

## Abstract

Metabolic disorders have been established as major risk factors for ocular complications and poor vision. However, little is known about the inverse possibility that ocular disease may cause metabolic dysfunction. To test this hypothesis, we assessed the metabolic consequences of a robust dietary challenge in several mouse models suffering from retinal mutations. To this end, mice null for melanopsin (*Opn4^-/-^*), the photopigment of intrinsically photosensitive retinal ganglion cells (ipRGCs), were subjected to five weeks of a ketogenic diet. These mice lost significantly more weight than wild-type controls or mice lacking rod and cone photoreceptors (*Pde6b^rd1/rd1^*). Although ipRGCs are critical for proper circadian entrainment, and circadian misalignment has been implicated in metabolic pathology, we observed no differences in entrainment between *Opn4^-/-^* and control mice. Additionally, we observed no differences in any tested metabolic parameter between these mouse strains. Further studies are required to establish the mechanism giving rise to this dramatic phenotype observed in melanopsin-null mice. We conclude that the causality between ocular disease and metabolic disorders merits further investigation due to the popularity of diets that rely on the induction of a ketogenic state. Our study is a first step toward understanding retinal pathology as a potential cause of metabolic dysfunction.

## Introduction

Twenty-eight million people worldwide suffer diminished vision from diabetic retinopathy.[1] Additionally, obesity leads to other ocular complications including macular degeneration, increased intraocular pressure, and cataracts.[2] These maladies of the eye are destined to increase as the prevalence of obesity increases in developed and developing countries. Although the association between metabolic disorders and ocular health are well established, in many such associations causality has not been established and mechanisms of pathogenesis remain to be determined. While it has been assumed that metabolic disorders cause ocular disease, one must consider the possibility that, in some cases, ocular disease may *lead* to systemic metabolic dysfunction. To test this possibility, we subjected a mouse model lacking a retinal photopigment to a robust dietary challenge and evaluated whole body metabolism.

*Opn4* ^*-/-*^ mice lack the photopigment melanopsin.[3] This photopigment renders a subset of retinal ganglion cells directly sensitive to light.[4] These intrinsically photosensitive retinal ganglion cells (ipRGCs) play a critical role in nonvisual, non-image forming (NIF) responses to light, such as the pupillary light reflex and circadian photoentrainment.[5] Most NIF responses are mediated through autonomic innervation. The photic regulation of the pineal’s production of melatonin is exclusively sympathetic.[6, 7] Dynamic control of pupillary aperture employs antagonistic influences from the sympathetic and parasympathetic components of the autonomic nervous system.[8] Presumably, the acute increases observed in heart rate and body temperature in response to light are also autonomically controlled.[9] The prominent regulatory function of ipRGCs in these diverse autonomic responses suggests that ipRGCs may play a broader role in autonomic physiology, perhaps even mediating light’s effect on general sympathetic tone.

Metabolism is also autonomically regulated. Although lipolysis in white adipose tissue (WAT) is controlled through humoral signals such as adipokines, insulin, and adrenal cathecholamines, within the past decade it has become increasingly clear that sympathetic innervation of WAT depots also plays a significant role in regulating lipolysis.[10] For example, the switch from carbohydrate to triglyceride metabolism in response to fasting is sympathetically regulated.[11] Because the NIF photoreceptive system regulates other autonomically driven responses, we tested whether impairment of ipRGCs, which mediate most NIF responses to light, impacts metabolism. To this end, we analyzed the metabolic phenotype of *Opn4* ^*-/-*^ mice maintained on a ketogenic diet, a robust dietary challenge that minimizes carbohydrate and protein intake. Because rod and cone pathways also indirectly signal through ipRGCs, mice lacking rods and cones (*Pde6b* ^*rd1/rd1*^), melanopsin-null mice lacking rods and cones (*Opn4* ^*-/-*^ *;Pde6b* ^*rd1/rd1*^), and wild-type mice were also challenged and assessed.

## Materials and Methods

### Animals

Experimental animals were adult male mice, 70–84 days old at the beginning of the experiments. Animals were derived either from an in-house breeding colony at the University of Virginia or purchased from Jackson Labs (Bar Harbor, ME). The following genotypes were used: *Opn4* ^*-/-*^ (mice null for melanopsin), [3], *Pde6b* ^*rd1/rd1*^ (mice homozygous for the *rd1* allele of the gene encoding the β-subunit of rod-specific phosphodiesterase which results in the degeneration of rods and cones)[12, 13], *Opn4* ^*-/-*^ *; Pde6b* ^*rd1/rd1*^ (a cross of the two aforementioned genotypes), and wild-type controls. All genotypes were congenic on the C57BL6/J background. Prior to experimentation, mice were maintained in standard animal housing conditions, under a 12h:12h light:dark cycle and fed standard chow (Harlan Teklad, Madison, WI; catalog # 8664) *ad libitum*. All animal care was conducted under the lighting condition to which the animals were being exposed at the time of care. Care under dark conditions was done under infrared illumination using infrared viewers.

Experimental procedures were carried out in accordance with the Association for Assessment and Accreditation of Laboratory Animal Care policies and approved by the University of Virginia Institutional Animal Care and Use Committee.

### Dietary Challenge

Age- and weight-matched wild-type, *Opn4* ^*-/-*^, *Pde6b* ^*rd1/rd1*^, and *Opn4* ^*-/-*^ *; Pde6b* ^*rd1/rd1*^ mice were transferred from standard housing to individual cages within light-tight boxes equipped with light emitting diodes (LEDs, Superbright LEDs, St. Louis, MO). Animals were maintained for five weeks in one of the following light regimens: 12h:12h blue light:dark (blue LD),12h:12h red light:dark (red LD), or constant darkness (DD). To ensure repeatability, we limited our light treatments to spectrally defined blue and red stimuli as opposed to undefined white light.

The irradiance of the blue (peak wavelength 468 nm, 20 nm half-peak bandwidth) or red (peak wavelength 635 nm, 20 nm half-peak bandwidth) portions of the light cycles were adjusted, through the use of neutral density filters (Lee Filters, Burbank, CA), to 3 x 10^11^ photons•s^-1^•cm^-2^, as measured at the center of each cage. While in light-tight boxes, animals were provided with water and a ketogenic diet (Bioserv, Frenchtown, NJ; catalog # F3666) *ad libitum*. The composition of the diet was 78.9% fat, 9.5% protein, and 0.76% carbohydrate and supplemented by the vendor with a vitamin and mineral mix to provide a diet complete in micronutrients. Mice were weighed every other day, and weight change was calculated as a percentage of the animal’s initial weight (weight on day 1). Repeated measures analysis of variance (RM-ANOVA) followed by the Tukey-Kramer *post-hoc* test was done using a statistics software package (SAS 9.1) to determine significant differences of percent weight change between groups.

### Circadian Entrainment

Wild-type and *Opn4* ^*-/-*^ mice were individually housed in cages contained within light-tight boxes and maintained under a 12h:12h white fluorescent light:dark cycle (white LD; 125 μW/cm^2^). Animals had *ad libitum* access to water and standard chow. After one week, animals were anesthetized with isoflurane and a sterilized transponder (G2 E-mitter Transponder, Mini Mitter/Respironics, Bend, OR) was surgically implanted into the abdominal cavity. Ketoprofen (i.p. at 0.1 mL/ 25 g) was administered post-surgically and wound clips were removed after 7–14 days. Following surgery, animals remained in the white LD cycle and continued on *ad libitum* water and standard chow. Each cage was equipped with a receiver (ER4000 Receiver, Mini Mitter, Philips Respironics, Bend, OR) and body temperature and activity were recorded every minute for the remainder of the experiment using Vitalview software. After two-weeks under a white LD cycle, both genotypes were placed under either a red LD cycle or blue LD cycle for a minimum of five weeks. At the time of the light cycle change, the normal chow diet was exchanged for the ketogenic diet made available *ad libitum*.

### Metabolic Monitoring of Mice

*Opn4* ^*-/-*^ and age-matched wild-type mice were maintained on normal chow for three days on either a 14h:10h red LD or 14h:10h blue LD cycle in Oxymax metabolic chambers (Columbus Instruments, Columbus, OH). After this “habituation run”, animals were returned to polystyrene cages under the same LD cycle where they remained on normal chow for four days. On the eighth day, animals were returned to the metabolic chambers under their designated LD cycle and were introduced to the previously described ketogenic diet. This period of time was designated as the “data run” and lasted for three days. Food and water intake, activity, oxygen consumption and carbon dioxide production were simultaneously monitored. Respiratory exchange ratios and heat production were automatically calculated from these parameters. Only the data from the last three days were used for statistical comparisons (t-tests).

## Results

### Light Condition and Genotype Affect Weight Change Upon a Dietary Challenge

**Fig 1:**
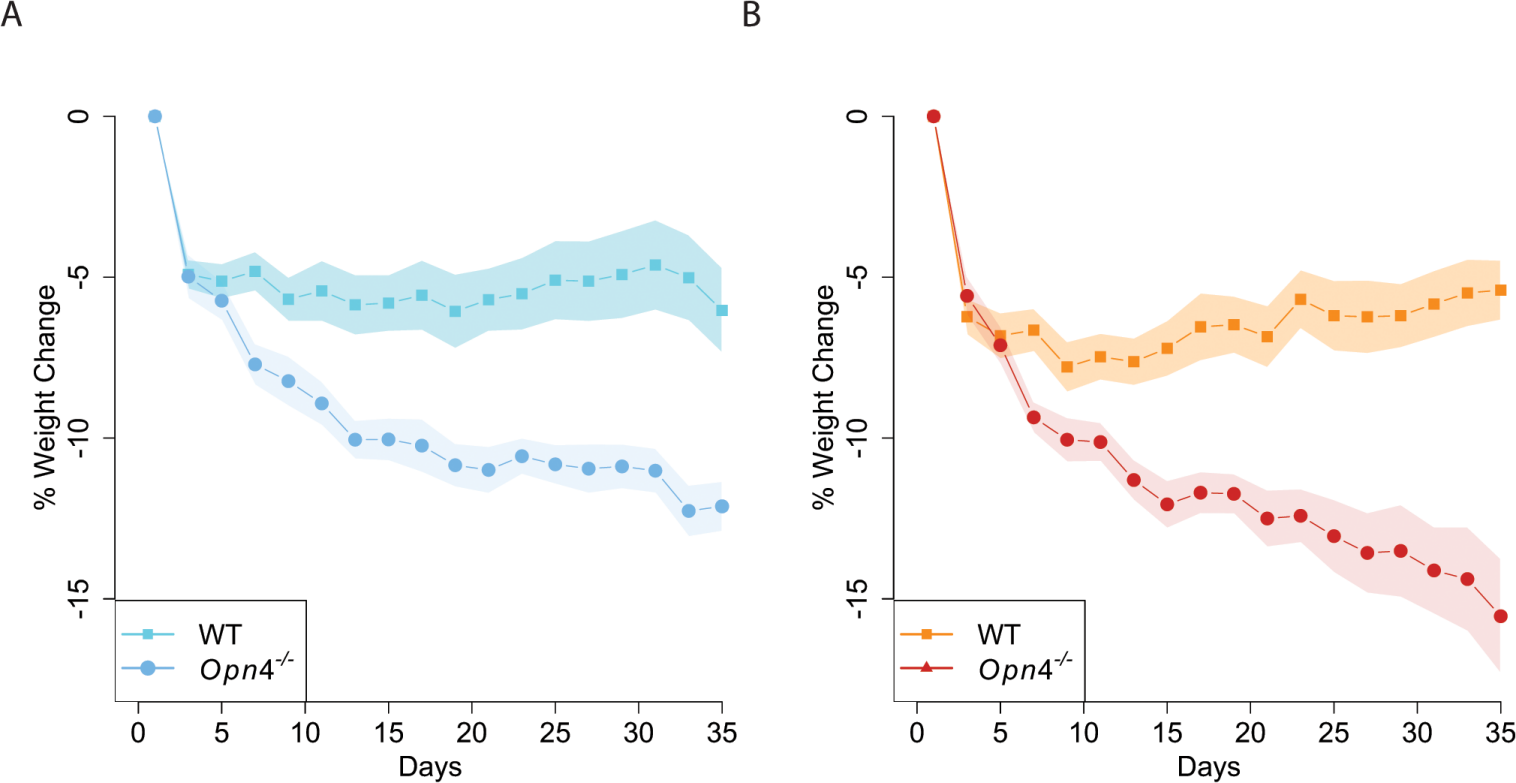
*Opn4* ^*-/-*^ mice lose significantly more weight as compared to the WT controls over the course of 5 weeks of ketogenic diet exposure A) under a 12h:12h blue LD and B) under a 12h:12h red LD cycle. RM-ANOVA followed by Tukey-Kramer post hoc analysis: *Opn4* ^***-/-***^ vs. WT in blue LD p = 0.0009; in red LD p = 0.0005. (*Opn4* ^***-/-***^ vs. WT at the end of week 2: in blue LD p = 0.0002, in red LD p = 0.0009; end of week 3: in blue LD p = 0.0001; in red LD p = 0.0003; end of week 4: in blue LD p<0.0001, in red LD p<0.0001; end of week 5: in blue LD p<0.0001, in red LD p<0.0001. For the weekly contrasts, alpha value was adjusted to 0.0017—Dunn-Sidak correction for contrasts. Under both blue and red LD cycles: *Opn4* ^***-/-***^ n = 9, WT n = 12)

**Fig 2:**
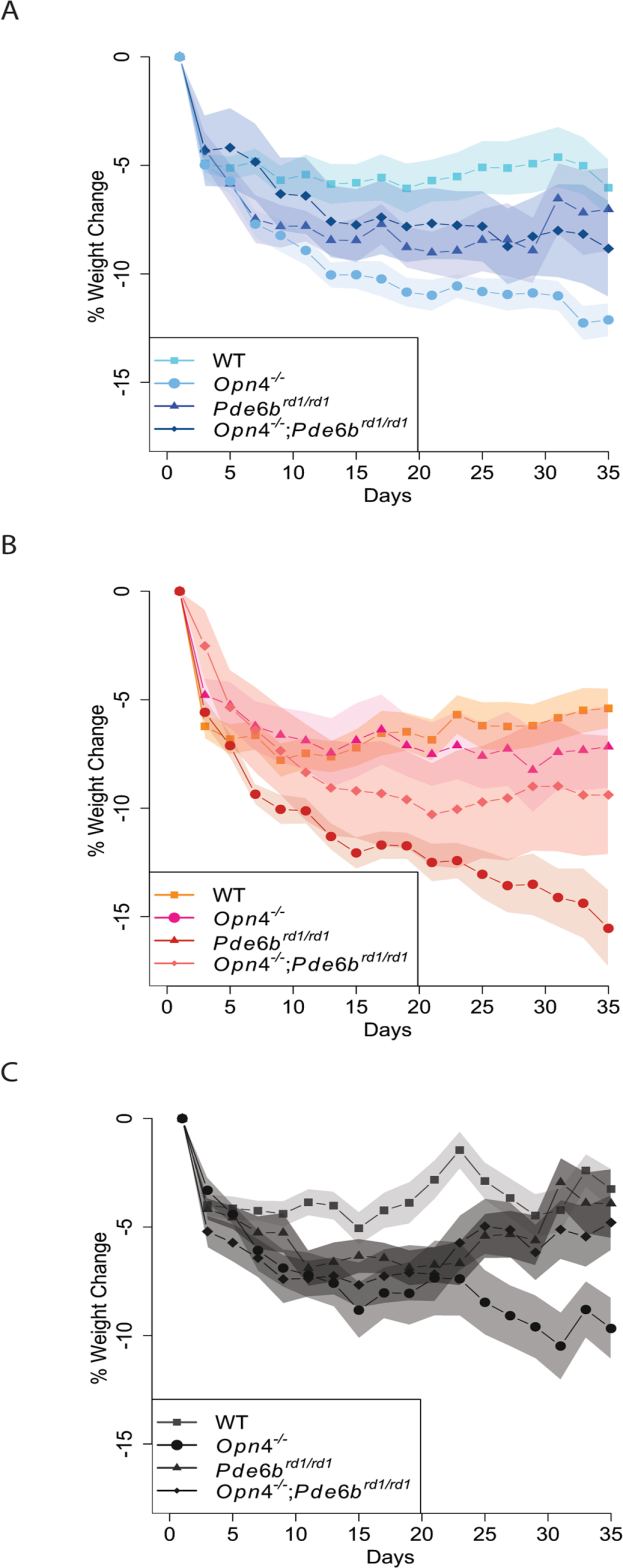
Analysis of all genotypes and light groups reveals significant effects of light regime (p<0.01) and genotype (p<0.0001) on weight change. Several genotypes and light groups demonstrate pronounced differences in the second half of the experiment. Overall analysis indicates *Opn4* ^***-/-***^ mice are significantly different in their weight change pattern than WTs (p<0.0001), but they are also different from *Opn4* ^***-/-***^ *; Pde6* ^***rd1/rd1***^ animals (p<0.05). Red LD condition causes more weight change than constant darkness over 5 weeks on the ketogenic diet challenge (p<0.01). A week by week analysis reveals the following differences in addition to the ones already mentioned in the legend of Fig 1: A) All genotypes under 12h:12h blue LD condition (end of week 2: WT vs *Opn4* ^***-/-***^, p = 0.0002; end of week 3: WT vs *Opn4* ^***-/-***^, p = 0.0001; end of week 4 and 5: WT vs *Opn4* ^***-/-***^, p<0.0001). B) All genotypes under 12h:12h red LD condition (end of week 4: *Opn4* ^***-/-***^ vs. *Opn4* ^***-/-***^ *; Pde6* ^***rd1/rd1***^, p = 0.001; end of week 5: *Opn4* ^***-/-***^ vs. *Opn4* ^***-/-***^ *; Pde6* ^***rd1/rd1***^, p<0.0001; *Opn4* ^***-/-***^ vs. *Pde6b rd1/rd1*, p = 0.0002). C) All genotypes in constant darkness (end of week 4: *Opn4* ^***-/-***^ vs. WT, p = 0.0016; end of week 5: *Opn4* ^***-/-***^ vs. WT, p = 0.0002). *Opn4* ^***-/-***^ mice in constant darkness lose significantly less weight than the ones in red LD by the end of week 5 (p = 0.0004). RM-ANOVA, followed by Tukey-Kramer *post hoc* analysis. (For the weekly contrasts, alpha value was adjusted to 0.0017—Dunn-Sidak correction for contrasts. Fig 2 includes data replotted from Fig 1 to demonstrate the full effects of all genotypes and light conditions. Under both blue and red LD cycles: *Opn4* ^***-/-***^ n = 9, WT n = 12, *Pde6* ^***rd1/rd1***^ n = 7, *Opn4* ^***-/-***^ *; Pde6* ^***rd1/rd1***^ n = 5. Under constant darkness: *Opn4* ^***-/-***^ n = 8, WT n = 8, *Pde6* ^***rd1/rd1***^ n = 8, *Opn4* ^***-/-***^ *; Pde6* ^***rd1/rd1***^ n = 6.)

In mice fed a ketogenic diet (KD), we observed significant effects of light condition (p = 0.0056) and genotype (p<0.0001) on weight change (Figs 1 and 2) by RM-ANOVA. *Opn4* ^*-/-*^ mice exhibited greater weight loss than WT mice over the course of 5 weeks in blue or red LD conditions (Fig 1A and 1B), and in the dark (Fig 2C). This result indicates that loss of melanopsin is linked to a weight loss phenotype in *Opn4* ^*-/-*^ mice on a C57BL6/J background. *Opn4* ^*-/-*^ mice not only lose more weight, but they also do not exhibit the weight change pattern seen in WT mice. WT mice in response to a KD typically lose weight, much of which is likely to be glycogen-associated water. [14, 15] However, in contrast to *Opn4* ^*-/-*^ mice, they gain back a portion of that weight after about two weeks of the dietary challenge, reaching equilibrium at a slightly lesser weight than the pre-challenge weight.

The Tukey-Kramer post-hoc test on general genotype effects over the entire 5 weeks of experiments revealed not only a significant difference between *Opn4* ^*-/-*^ mice and their WT controls (p<0.0001), but also between *Opn4* ^*-/-*^ mice and the *Opn4* ^*-/-*^ *; Pde6b* ^*rd1/rd1*^ animals (p = 0.0211) (Fig 2). The latter difference suggests a rescue of the extreme weight loss phenotype by the loss of rods and cones in the retina, and will be discussed further. Among all genotypes, weight change patterns are significantly different between red LD and constant darkness (p = 0.0040), whereas there is no difference between blue LD and constant darkness (Fig 2B and 2C). Constant darkness represents one condition where animals lose less weight than in other light conditions. In effect, constant darkness appears to confer partial protection against the dietary challenge.

### Initial Differences in Weight Change Patterns Become Exaggerated Over Time

Light (p<0.0001) and genotype (p<0.0001) are both highly significantly affecting weight change over time. Fig 1 reveals a difference in the profiles of weight change, especially during the second phase of the dietary challenge when WT animals stabilize. Due to this difference in weight loss profiles of the light and genotype interaction, we compared groups at regular time intervals over the five weeks course of the experiments. Specifically, all groups were compared to each other by the end of each week.

Consistent with the fact that the *Opn4* ^*-/-*^ mice do not stabilize their weight after two weeks on the KD, the difference between *Opn4* ^*-/-*^ mice and WT controls becomes significant as early as the end of the second week under blue LD (p = 0.0002; Figs 1A and 2A) and under red LD (p = 0.0009; Figs 1B and 2B), and continues to be so until the end of week five, in both red and blue LD conditions (week 3: p = 0.0001 in blue LD, p = 0.0003 in red LD; weeks 4 and 5: p<0.0001 in both blue and red LD). *Opn4* ^*-/-*^ mice differ from WT mice in the dark by the end of fourth (p = 0.0016) and fifth (p = 0.0002) weeks, indicating a strong genotype effect independent of light exposure (Fig 2C). Furthermore, *Opn4* ^*-/-*^; *Pde6b* ^*rd1/rd1*^ mice are significantly different than *Opn4* ^*-/-*^ mice in red LD by the end of the 4^th^ (p = 0.0010) and 5^th^ (p<0.0001) weeks (Fig 2B). The end of 5^th^ week is also when *Pde6b* ^*rd1/rd1*^ mice differ from *Opn4* ^*-/-*^ mice in red light (p = 0.0002) (Fig 2B).

According to this week by week analysis, the only specific time where a significant difference is seen between light conditions is by the end of week 5, between *Opn4* ^*-/-*^ mice in red LD and constant dark (p = 0.0004) (Fig 2B and 2C) (Note that for all weekly comparisons, alpha value has been adjusted to 0.0017—Dunn-Sidak correction for contrasts).

### Circadian Deficits Cannot Account for the Observed Weight Change Phenotype

**Fig 3:**
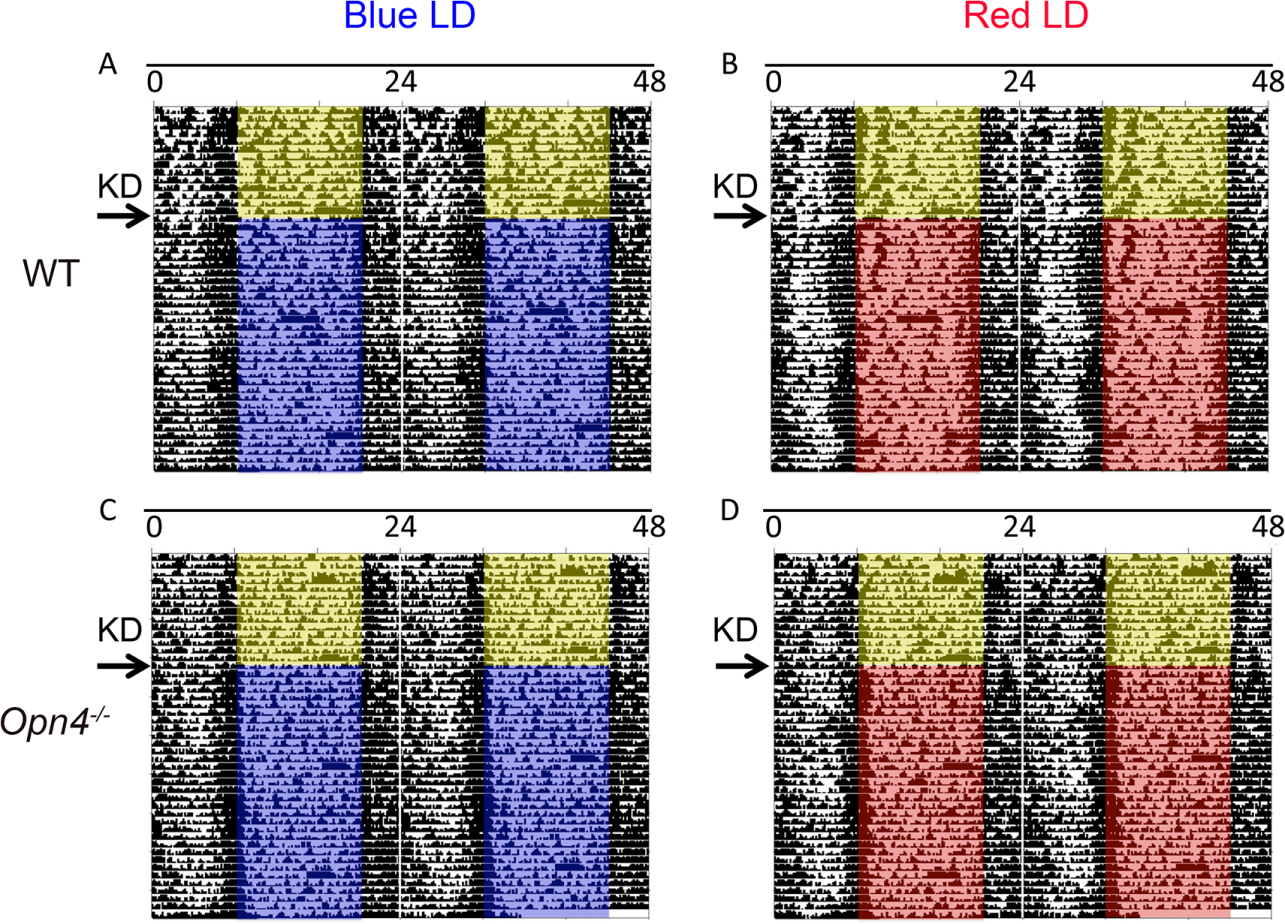
Both *Opn4* ^*-/-*^ mice and the wild type controls entrain to a 12h:12h fluorescent LD cycle (yellow block corresponds to fluorescent lights on) as measured by radio-telemetry. After two weeks, animals are transferred to a 12h:12h blue or red LD cycle (red and blue blocks corresponds to lights on) and concurrently placed on a KD. A and B) Representative actograms of WT animals. C and D) Representative actograms of *Opn4* ^***-/-***^ animals. Arrows indicate the day animals are switched to the KD. All actograms have been double-plotted with a 10-minute bin size. (Under red LD cycle: *Opn4* ^***-/-***^ n = 4, WT n = 5. Under blue LD cycle: *Opn4* ^***-/-***^ n = 4, WT n = 4.)

**Fig 4:**
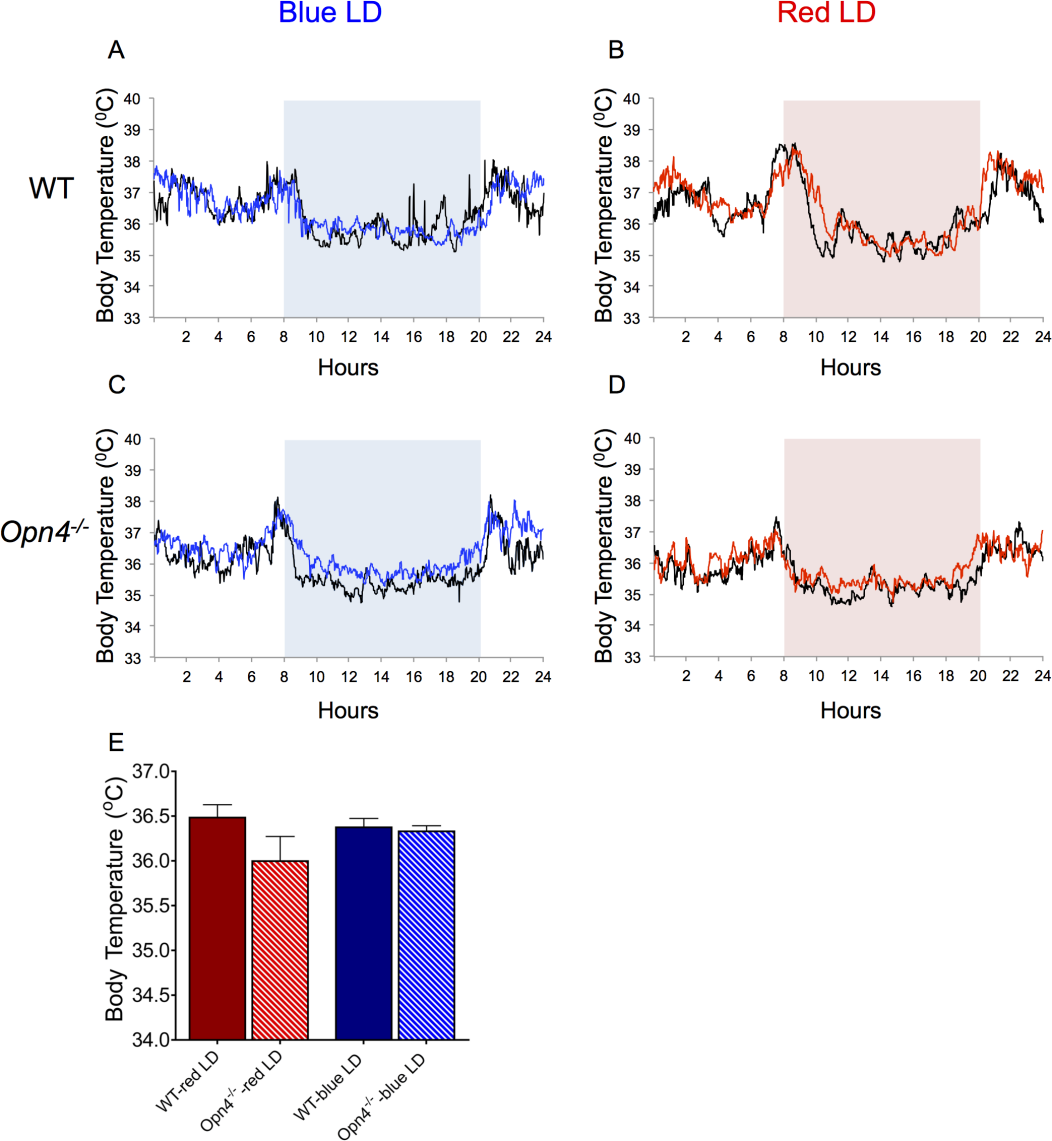
Animals assessed for entrainment as in Fig 3 were simultaneously assessed for body temperature by radio-telemetry. *Opn4* ^***-/-***^ mice, like WT animals, can entrain their body temperature rhythms to both red and blue LD cycles. Black traces represent one day temperature records from animals 10 days prior to KD challenge and concurrent transfer to a red or blue LD cycle. Colored traces represent one day temperature records from animals 10 days after the initiation of KD challenge and concurrent transfer to a red or blue LD cycle. Rectangles correspond to the time during which lights were on. All animals were entrained to all light conditions, exhibiting lower body temperatures throughout the light portion of the day, and peak temperatures around lights on and off. A) Wild type, before (black) and after (blue) blue LD exposure. B) Wild type, before (black) and after (red) red LD exposure. C) *Opn4* ^***-/-***^, before (black) and after (red) red LD exposure. D) *Opn4* ^***-/-***^, before (black) and after (red) red LD exposure. E) *Opn4* ^***-/-***^ animals did not have significantly different average body temperatures than their wild type controls in either blue or red LD cycles. All values represent group means with ±SEM. (t-tests used to compare average temperatures in each light and genotype group. Under red LD cycle: *Opn4* ^***-/-***^ n = 4, WT n = 5. Under blue LD cycle: *Opn4* ^***-/-***^ n = 4, WT n = 4.)

To test whether circadian deficits can account for the body mass phenotype observed in response to ketogenic diet challenge, we assessed entrainment in *Opn4* ^*-/-*^ and WT mice. After two weeks entrainment to a 12h:12h white fluorescent LD cycle, animals were concomitantly placed on a KD and under a blue or red LD cycle. Both red and blue LD cycles at the tested intensities are capable of entraining *Opn4* ^*-/-*^ and WT mice fed a KD. Locomotor activity rhythms of both genotypes remained synchronized to the LD cycle (Fig 3). All animals exhibited periods of 24 hours under white fluorescent LD or blue or red LD cycles (Chi square periodogram analysis, ClocksLab, data not shown). WT and *Opn4* ^*-/-*^ animals also displayed diurnally rhythmic body temperatures that peaked at the light-to-dark and dark-to-light transitions before and after the lighting and dietary switch. Body temperature rhythms of WT (Fig 4A and 4B) and *Opn4* ^*-/-*^ mice also remained synchronized to the LD cycle after the switch from fluorescent light to blue or red LD (Fig 4C and 4D).

Mean body temperatures before and after the light/diet switch were calculated in order to address any differences between groups. Body temperatures of *Opn4* ^*-/-*^ mice were similar before and after the consumption of the KD in either a red or blue LD cycle (Fig 4E). This was also the case in WT mice. Taken together, these results indicate that the weight change differences observed between the genotypes are not due to variations in body temperature or an inability to synchronize with the environmental light-dark cycle.

### Food Intake, Activity, and Metabolic Rate of Opn4^-/-^ Mice Did Not Differ from WT

**Fig 5:**
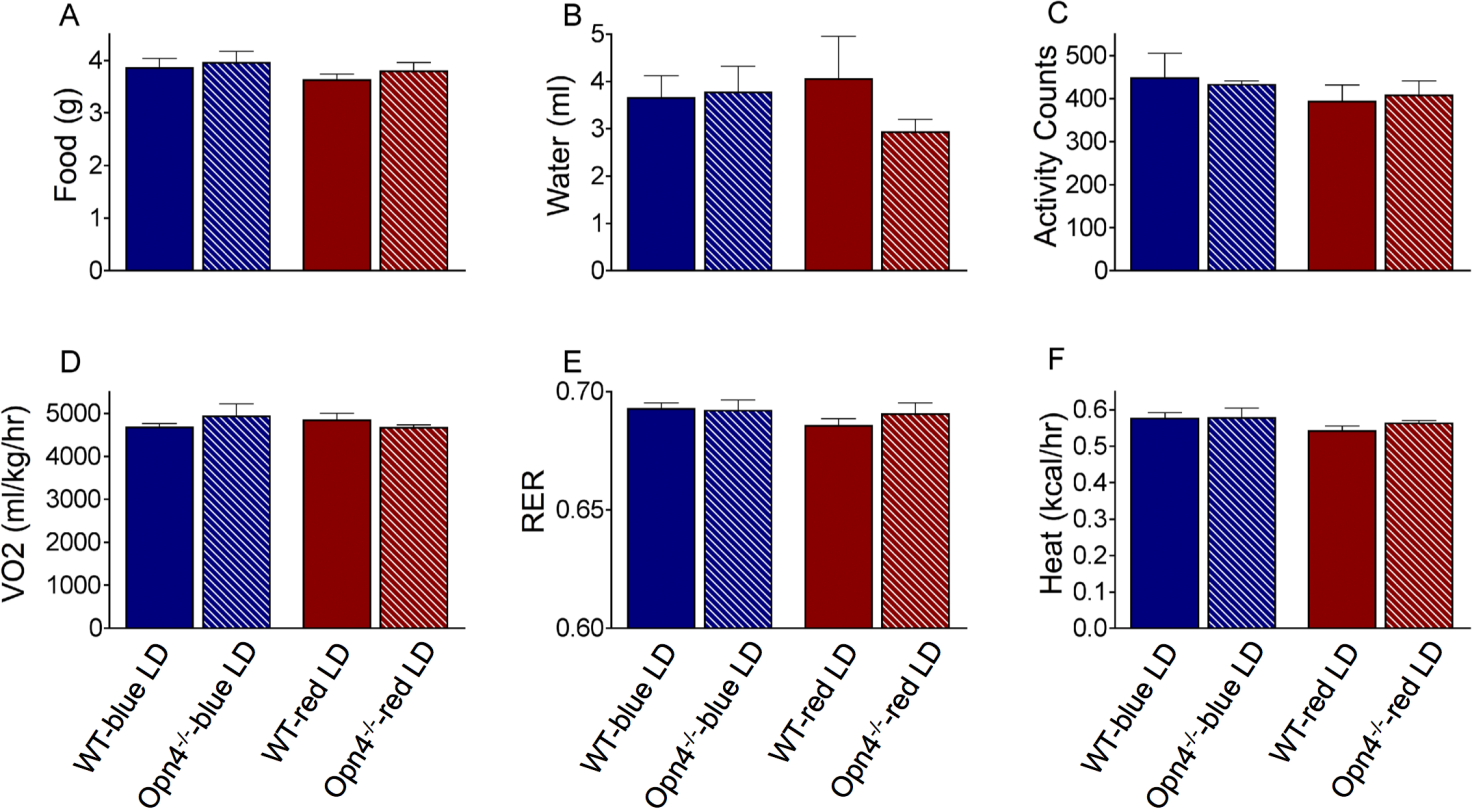
The extreme weight loss profile in *Opn4* ^*-/-*^ mice could not be attributed to any gross-metabolic changes that occur early during the ketogenic diet—blue or red LD challenge. Solid bars stand for WT animals, striped bars stand for *Opn4* ^***-/-***^ mice. Bars are also color-coded: blue stands for blue LD, red stands for red LD. All mice were fed *ad libitum* ketogenic diet during the course of the experiment (n = 4 mice / group). All groups, regardless of their genotype and the light conditions they were in, consumed similar amounts of food (A) and water (B) (t-tests: p>0.05). Activity was measured as infrared beam crossings and expressed as number of counts per 15 minute intervals. C) Activity levels were similar in all groups (t-test: p>0.05). There were no significant differences between groups in metabolic parameters such as oxygen consumption (D) and respiratory exchange rate (RER) (E). Respiratory exchange rate was calculated for each animal by taking the carbon dioxide production / oxygen consumption ratio. F) Heat production also did not differ significantly between groups. All values represent group means with ±SEM. (Under red and blue LD cycle: *Opn4* ^***-/-***^ n = 4, WT n = 4)

Several gross metabolic outputs were measured in WT and *Opn4* ^*-/-*^ mice while they were challenged with a KD. The extreme weight loss phenotype observed in *Opn4* ^*-/-*^ mice is not due to food consumption (Fig 5A), changes in activity (Fig 5C), metabolic rate (Fig 5D) or heat production (Fig 5F). *Opn4* ^*-/-*^ mice were also not generating excess amounts of heat. In addition, all groups consumed equivalent amounts of the KD, and showed similar metabolic rates. Respiratory exchange ratios also did not differ between groups. To summarize, the excessive weight loss observed in *Opn4* ^*-/-*^ mice could not be attributed to any changes in the metabolic parameters of the animals. Although this seems to be an unexpected phenotype for the *Opn4* ^*-/-*^ mice, it must be noted that due to technical reasons, the metabolic experiments could only capture an early (during the first two weeks of KD) and short (3 days) time window of KD challenge, which lasted for five weeks in the actual weight loss study. It should also be noted that the more pronounced differences in weight change between *Opn4* ^*-/-*^ and WT mice occur after 2 weeks of KD exposure, according to the time-point analysis (see previous headline). A future study will investigate the gross metabolic differences between the two genotypes should thus focus on later time points.

## Discussion

It is difficult to conceive how a mutation that eliminates a retinal photopigment can produce such a profound whole-body metabolic phenotype in response to dietary challenge. The observed differences in body weight could be a consequence of differences between the genotypes in caloric intake or caloric expenditure. However, we observed no difference in food consumption or activity levels between genotypes or among the different lighting conditions (Fig 5A and 5C). Additionally, none of the metabolic metrics showed any differences between the melanopsin-null and wild-type mice (Fig 5). We assessed metabolic parameters immediately upon challenging the animals with the ketogenic diet. The weight loss phenotype is manifested after approximately three days on the ketogenic diet, however, potential metabolic differences may become apparent only after several days of dietary challenge.

Connections are emerging between circadian dysfunction and metabolic pathology.[16] Because melanopsin-expressing ipRGCs play a role in synchronizing circadian rhythms to the astronomical day, the metabolic phenotype could arise from an insufficiency in photoentrainment. However, we observed no differences in diurnal rhythms of activity or body temperature among our experimental groups (Fig 5), so the root cause of the phenotype remains unknown.

A photopigment deficit suggests that the diet-induced phenotype would be light dependent. For this reason, we analyzed our animals under blue:dark or red:dark LD cycles. Because melanopsin is optimally sensitive to blue light relative to red light, we would expect to see light dependent effects vary as a function of spectra. While we observe a more robust phenotype in animals maintained under red:dark light cycles vs. blue:dark light cycles, as would be expected in mice lacking the blue-sensitive melanopsin photopigment, the weight loss phenotype is also observed in animals maintained under constant darkness. Accordingly, we have concluded that the phenotype is derived from light-dependent effects revealed by our light treatments and light-independent effects of genotype (Fig 1).

How can a partially light-independent phenotype arise from a photopigment mutation? There are several possibilities. In the postnatal retina, melanopsin-driven photoresponses in ipRGCs occur before rod and cone responses develop.[17] ipRGC signaling is not only critical for the proper development and wiring of the retina, but also mediates a fetal light response that regulates eye development.[18, 19] Melanopsin is also required for forming proper connections to central retinorecipient sites.[20] We postulate that loss of melanopsin may interfere with proper light-dependent retinal innervation of hypothalamic targets, especially those implicated in energy homeostasis. However, the aberrant circuitry laid down in a light-dependent manner during neonatal development may cause homeostatic differences in basal metabolic rates that are light-independent in the adult.

An alternate hypothesis is that melanopsin is functioning as sensor for something other than light, perhaps nutrients. *Drosophila* rhodopsin can function as a thermoreceptor in a light-independent manner.[21] Interestingly, flies null for rhodopsin that exhibit deficient thermosensitivity can be rescued by targeting expression of melanopsin to the omatidial photoreceptors. In our studies, wild-type mice appear to sense the dietary challenge and then adapt metabolically to preserve body weight. By contrast, melanopsin-null mice fail to adapt to the dietary challenge, suggesting the ketogenic insult is going undetected, thereby resulting in no metabolic accommodation. If melanopsin were playing a nonphotic function as a nutrient sensor necessary for metabolic adaptation, then melanopsin-null mice would exhibit the weight loss phenotype we observe.

A third hypothesis is that the deletion at the melanopsin locus is potentially altering expression of a metabolic gene in the same genomic region, either by direct gene-deletion or modification of a critical enhancer element. However, it should be noted that there are no known metabolic genes or enhancer/repressor elements near the modified locus. Additionally, *Opn4* ^*-/-*^ *; Pde6b* ^*rd1/rd1*^ animals exhibit a rescue phenotype over the *Opn4* ^*-/-*^ mice. Taken together, it seems unlikely that the observed phenotype is an epiphenomenon of the targeted gene deletion.

The mechanism responsible for the weight loss phenotype in melanopsin-deficient mice remains unknown. It is accepted that the autonomic nervous system exerts partial control over adiposity of fat depots. We propose that the visual photoreceptors (rods and cones) and the ipRGCs antagonistically influence the autonomic nervous system, either acutely, resulting in the observed light-dependent effects, or during development, resulting in our observed light-independent weight loss pattern. This model is based on the surprising observation that elimination of the visual photoreceptors, as is the case in *Pde6b* ^*rd1/rd1*^ mice, rescues the weight-loss phenotype experienced by melanopsin-null mice. (Fig 2) Further studies are needed to test this proposed “push-pull” model.

We have shown that mice deficient in the retinal photopigment melanopsin exhibit extreme weight loss when challenged with a ketogenic diet. It has been reported that C57BL6/J mice maintained on a KD lose lean mass but protect white adipose tissue stores.[22] The difference observed in body mass between our wild-type and melanopsin-null mice may arise from a difference in fat metabolism, where wild-type mice lose lean mass while *Opn4* ^*-/-*^ mice lose fat mass in addition to lean mass. Body composition studies are underway to address this possibility.

Ketogenic diets have been used to treat refractory epilepsy in children, and many popular low-carbohydrate diets are ketogenic during the initial induction phase. Because of the popularity of such diets, understanding the consequences of such dietary challenge, especially in those who suffer from retinal disease, is critical. Our study is a first step toward this goal.

## References

[1] Abcouwer SF, Gardner TW. Diabetic retinopathy: loss of neuroretinal adaptation to the diabetic metabolic environment Annals of the New York Academy of Sciences. 2014;1311:174–90. 10.1111/nyas.12412 PubMed 24673341 PMC4154702

[2] Cheung N, Wong TY. Obesity and eye diseases. Survey of ophthalmology. 2007;52(2):180–95. 10.1016/j.survophthal.2006.12.003 PubMed 17355856 PMC2698026

[3] Panda S, Sato TK, Castrucci AM, Rollag MD, DeGrip WJ, Hogenesch JB, et al Melanopsin (Opn4) requirement for normal light-induced circadian phase shifting. Science. 2002;298(5601):2213–6. PubMed . 12481141 10.1126/science.1076848

[4] Lucas RJ, Hattar S, Takao M, Berson DM, Foster RG, Yau KW. Diminished pupillary light reflex at high irradiances in melanopsin-knockout mice. Science. 2003;299(5604):245–7. PubMed . 12522249 10.1126/science.1077293

[5] Do MT, Yau KW. Intrinsically photosensitive retinal ganglion cells. Physiol Rev. 2010;90(4):1547–81. PubMed 10.1152/physrev.00013.2010 20959623 PMC4374737

[6] Klein DC, Weller JL, Moore RY. Melatonin metabolism: neural regulation of pineal serotonin: acetyl coenzyme A N-acetyltransferase activity. Proc Natl Acad Sci U S A. 1971;68(12):3107–10. PubMed . 4332009 10.1073/pnas.68.12.3107 PMC389600

[7] Lingappa JR, Zigmond RE. Limited recovery of pineal function after regeneration of preganglionic sympathetic axons: evidence for loss of ganglionic synaptic specificity. J Neurosci. 2013;33(11):4867–74. PubMed 10.1523/JNEUROSCI.3829-12.2013 23486957 PMC3640627

[8] Neuhuber W, Schrodl F. Autonomic control of the eye and the iris. Auton Neurosci. 2011;165(1):67–79. PubMed 10.1016/j.autneu.2010.10.004 21071284

[9] Cajochen C, Munch M, Kobialka S, Krauchi K, Steiner R, Oelhafen P, et al High sensitivity of human melatonin, alertness, thermoregulation, and heart rate to short wavelength light. J Clin Endocrinol Metab. 2005;90(3):1311–6. PubMed . 15585546 10.1210/jc.2004-0957

[10] Bartness TJ, Shrestha YB, Vaughan CH, Schwartz GJ, Song CK. Sensory and sympathetic nervous system control of white adipose tissue lipolysis. Mol Cell Endocrinol. 2010;318(1–2):34–43. PubMed 10.1016/j.mce.2010.01.028 19747957 PMC2826518

[11] Izumida Y, Yahagi N, Takeuchi Y, Nishi M, Shikama A, Takarada A, et al Glycogen shortage during fasting triggers liver-brain-adipose neurocircuitry to facilitate fat utilization. Nature communications. 2013;4:2316 10.1038/ncomms3316 PubMed 23939267 PMC3753545

[12] Bowes C, Danciger M, Kozak CA, Farber DB. Isolation of a candidate cDNA for the gene causing retinal degeneration in the rd mouse. Proc Natl Acad Sci U S A. 1989;86(24):9722–6. PubMed . 2481314 10.1073/pnas.86.24.9722 PMC298573

[13] Pittler SJ, Baehr W. Identification of a nonsense mutation in the rod photoreceptor cGMP phosphodiesterase beta-subunit gene of the rd mouse. Proc Natl Acad Sci U S A. 1991;88(19):8322–6. PubMed . 1656438 10.1073/pnas.88.19.8322 PMC52500

[14] Kreitzman SN, Coxon AY, Szaz KF. Glycogen storage: illusions of easy weight loss, excessive weight regain, and distortions in estimates of body composition. The American journal of clinical nutrition. 1992;56(1 Suppl):292S–3S. PubMed . 1615908 10.1093/ajcn/56.1.292S

[15] MacKay EM, Bergman HC. The relationship between glycogen and water storage in the liver. J Biol Chem. 1932;96:373–80.

[16] Maury E, Hong HK, Bass J. Circadian disruption in the pathogenesis of metabolic syndrome. Diabetes & metabolism. 2014. 10.1016/j.diabet.2013.12.005 PubMed . 24433933

[17] Sekaran S, Lupi D, Jones SL, Sheely CJ, Hattar S, Yau KW, et al Melanopsin-dependent photoreception provides earliest light detection in the mammalian retina. Curr Biol. 2005;15(12):1099–107. 10.1016/j.cub.2005.05.053 PubMed . 15964274 PMC4316668

[18] Kirkby LA, Feller MB. Intrinsically photosensitive ganglion cells contribute to plasticity in retinal wave circuits. Proc Natl Acad Sci U S A. 2013;110(29):12090–5. 10.1073/pnas.1222150110 PubMed 23821744 PMC3718101

[19] Rao S, Chun C, Fan J, Kofron JM, Yang MB, Hegde RS, et al A direct and melanopsin-dependent fetal light response regulates mouse eye development. Nature. 2013;494(7436):243–6. 10.1038/nature11823 PubMed 23334418 PMC3746810

[20] Renna JM, Weng S, Berson DM. Light acts through melanopsin to alter retinal waves and segregation of retinogeniculate afferents. Nat Neurosci. 2011;14(7):827–9. 10.1038/nn.2845 PubMed 21642974 PMC3125440

[21] Shen WL, Kwon Y, Adegbola AA, Luo J, Chess A, Montell C. Function of rhodopsin in temperature discrimination in Drosophila. Science. 2011;331(6022):1333–6. 10.1126/science.1198904 PubMed . 21393546

[22] Kennedy AR, Pissios P, Otu H, Roberson R, Xue B, Asakura K, et al A high-fat, ketogenic diet induces a unique metabolic state in mice. Am J Physiol Endocrinol Metab. 2007;292(6):E1724–39. PubMed . 17299079 10.1152/ajpendo.00717.2006

